# Medical and Philosophical News

**Published:** 1781-04

**Authors:** 


At a public meeting of the Royal Medical Society of Paris on the 6th of March, at their apartments in the Louvre, M. Lorry read a diſcourſe on the odours of medicines, divided into five claſſes; M. Carrere communicated the plan of a new work on the mineral waters of France; M. Defourcroy delivered ſome obſervations on a new method of employing certain reagents in the analyſis of mineral waters; M.

Vicq.

Vicq. d'Azyr, ſecretary to the ſociety, pronounced the eulogium of the late M. Navier, phyſician at Chalons ſur Marne, and fellow of the ſociety; M. Caille gave an account of ſome chymical inquiries on the different proceſſes employed for preparing emetic tartar; the Abbé Teſſier read a paper on a diſeaſe which he calls "la maladie rouge," and which has lately proved fatal to a prodigious number of ſheep in the diſtrict of Sologne; the buſineſs of the meeting ended with the reading of a paper by M. Mauduyt on the effects of electricity, applied to incubation and vegetation.

